# Nutritional Value of *Moringa oleifera* Lam. Leaf Powder Extracts and Their Neuroprotective Effects via Antioxidative and Mitochondrial Regulation

**DOI:** 10.3390/nu13072203

**Published:** 2021-06-26

**Authors:** Elena González-Burgos, Isabel Ureña-Vacas, Marta Sánchez, M. Pilar Gómez-Serranillos

**Affiliations:** Department of Pharmacology, Pharmacognosy and Botany, Faculty of Pharmacy, Universidad Complutense de Madrid (UCM), 28040 Madrid, Spain; elenagon@ucm.es (E.G.-B.); isabelur@ucm.es (I.U.-V.); martas15@ucm.es (M.S.)

**Keywords:** *Moringa oleifera*, neurodegenerative disorders, neuroprotection, oxidative stress, nutrients

## Abstract

Age-related neurodegenerative disorders are an increasing public health problem. Oxidative stress is one of the major causes. Medicinal plant-based functional foods can be effective for these diseases. The aim of this work is to investigate the neuroprotective role of methanol extracts of *Moringa oleifera* leaf powder on antioxidant/oxidant imbalance and mitochondrial regulation in a H_2_O_2_-induced oxidative stress model in human neuroblastoma cells. On nutritional analysis, results showed that moringa contained 28.50% carbohydrates, 25.02% proteins, 10.42% fat, 11.83% dietary fiber, 1.108 mg β-carotene, 326.4 µg/100 g vitamin B1 and 15.2 mg/100 g vitamin C. In*-*vitro assays revealed that moringa methanol extracts had more phenolic content and higher antioxidant activity than acetone extracts. Moreover, pretreatments with methanol extracts showed a protective effect against H_2_O_2_-induced oxidative damage through increasing cell viability and reducing free radicals. Furthermore, the extract decreased lipid peroxidation and enhanced glutathione levels and antioxidant enzyme activity. Finally, moringa also prevented mitochondrial dysfunction by regulating calcium levels and increasing mitochondrial membrane potential. The most active concentration was 25 µg/mL. In summary, the nutritional and functional properties of *Moringa oleifera* as a neuroprotective agent could be beneficial to protect against oxidative stress and provide necessary nutrients for a healthy diet.

## 1. Introduction

Age-related neurodegenerative diseases such as Alzheimer’s and Parkinson’s disease are heterogeneous clinical diseases with a multi-causal origin. Neurodegenerative diseases affect millions of people, causing disability and great economic impact. Conventional treatments are symptomatic, and there are currently no drugs that cure any of these neurodegenerative diseases. In this context, medicinal plants are an inexhaustible source of molecules with pharmacological properties. The World Health Organization (WHO) and European Medicines Agency (EMA) reported several herbal products for the management of neurodegenerative diseases, such as folium *Ginkgo* [1,2]. Currently, research is aimed at finding new herbal products with protective and therapeutic properties against neurodegenerative disease. 

Oxidative stress is one of the major causes implicated in the pathogenic development of age-related neurodegenerative disorders. Oxidative stress occurs from an imbalance between reactive oxygen species (ROS) generation and antioxidant defense system. The oxidative stress theory of aging postulates that physical and functional losses in the aged are caused by ROS accumulation, their consequent reactivity and damage to cellular macromolecules [3]. Post-mortem brains of patients with neurodegenerative diseases have shown the presence of biomarkers of oxidative stress including protein carbonyl, *trans*-4-hydroxy-2-nonenal (4-HNE) and malondialdehyde (MDA) [4].

*Moringa oleifera* Lam. (Moringaceae family, commonly known as Horseradish tree) is a perennial herb tree that extensively grows in tropical and subtropical countries. *Moringa oleifera* is employed as vegetable, herbal tea and processed foods for its nutritional properties as a source of proteins and essential amino acids (i.e., cysteine, methionine, lysine and tryptophan) [5]. Moreover, the leaves of *Moringa oleifera* are rich in flavonoids, carotenoids and ascorbic acid [6]. Furthermore, this herbal product has demonstrated health benefits beyond its great nutritional value. Hence, in traditional medicine, ancient rules included the leaves of *Moringa oleifera* for its beneficial properties in mental health and smooth skin [7]. Moreover, *Moringa oleifera* leaves have been attributed numerous therapeutic applications in in-vitro and in-vivo studies such as antibacterial, antifungal, antiviral, cytotoxic, antihyperglycemic, antioxidant, anti-inflammatory, antiparasitic and cardioprotective activities [8]. In addition, there are several clinical studies on *Moringa oleifera* focused on its pharmacological role in metabolic syndrome, type 2 diabetes mellitus, osteoporosis, anemia and dyslipidemias [9]. However, despite its great nutritional and therapeutic value, *Moringa oleifera* leaves are not as popular as other leafy vegetables, and its potential pharmacological activities are still unexplored, particularly in terms of its role as neuroprotective agent. 

Considering the growing consumption of *Moringa oleifera* in the world, the aim of this work was to investigate the *Moringa oleifera* ability to prevent and treat hydrogen peroxide-induced oxidative stress in neuroblastoma cells. The effect of methanol extract of moringa leaf powder on antioxidant/ROS imbalance and mitochondrial regulation was investigated in the human SH-SY5Y cell line.

## 2. Materials and Methods

### 2.1. Preparation of Moringa oleifera Extracts 

Fresh leaves of *Moringa oleifera* were collected in Piribebuy, Paraguay between March 2016 and August 2016. After collection, the leaves were dried at room temperature and pulverized for further experimental analysis. Leaf powder of *Moringa oleifera* (over 50 mg) was macerated with methanol and acetone (2 mL) for 24 h at room temperature. The obtained extracts were then filtered through Whatman filter paper and evaporated until dry at room temperature. Extracts were kept in a freezer at −20 °C until use.

### 2.2. Nutritional Value

The nutritional value of *Moringa oleifera* was performed in triplicate to estimate fat, proteins, ash, carbohydrates, moisture, dietary fiber, energy, total carotenoids and vitamins B1 and C. The energy content in moringa was determined using Atwater factors. The protein content was determined based on the analysis of the total nitrogen content by the Kjeldahl method using a 6.25 correction factor [10]. Total available carbohydrates were analyzed based on an anthrone colorimetric technique; absorbance was measured at 630 nm [11]. Total dietary fiber content was quantified by the Association of Official Analytical Chemists (AOAC) enzymatic–non-gravimetric method (993.21) [10]. Moisture content was calculated by desiccation and ash content by combustion following the AOAC 984.25 method and 942.05 method, respectively [10]. Total fat was determined after petroleum ether extraction and consequent drying at 105 °C following the AOAC 983.23 procedure [10]. The total carotenoid content was determined by spectrophotometry (446 nm) according to the method described in AOAC [10]. The determination of vitamin B1 (thiamine), based on its oxidation to the fluorescent thiochrome with alkaline potassium hexacyanoferrate (III) (potassium ferricyanide), was performed following the AOAC 942.23 procedure [10]. Finally, vitamin C content was analyzed by high-performance liquid chromatography (HPLC) technique using Agilent 1260 equipment with a reversed-phase column and a diode array detector. The mobile phase was water acidified to pH 2.6 with sulfuric acid. The rate of flow was 0.9 mL/min. Detection wavelength for the UV-visible detector was set at 245 nm [12].

### 2.3. Total Phenolic Compounds

The Folin–Ciocalteu method was used to determine the total phenolic compounds presented in methanol and acetone extracts of moringa leaf powder. Extracts were incubated with Folin–Ciocalteu reagent (Sigma-Aldrich, St. Louis, MO, USA) for 5 min followed by 10% Na_2_CO_3_ incubation for 40 min. Absorbance was determined at 752 nm using a SPECTROstar BMG microplate reader (BMG Labtech Inc., Ortenberg, Germany) [13].

### 2.4. In-Vitro Antioxidant Assays

#### 2.4.1. 1,1-Diphenyl-2-picrylhydrazyl (DPPH) Method

Extracts of moringa were incubated with 1,1-Diphenyl-2-picrylhydrazyl (DPPH) (50 µM) (Sigma-Aldrich) for 30 min. Absorbance was then measured at 517 nm using a SPECTROstar BMG microplate reader [14].

#### 2.4.2. Oxygen Radical Absorbance Capacity (ORAC) Assay

Extracts of moringa were incubated with fluorescein (70 nm) for 10 min of darkness. Then, 2,2’-Azobis(2-amidinopropane) dihydrochloride (AAPH) solution (Sigma-Aldrich) was added to the 96-well plates. Fluorescence was recorded for 98 min at a 485 nm excitation wavelength and at a 520 nm emission wavelength in a FLUOstar OPTIMA fluorimeter (BMG Labtech, Ortenberg, Germany) [15].

#### 2.4.3. Ferric Reducing Antioxidant Power (FRAP) Assay

Extracts of moringa were incubated with FRAP reagent for 30 min. Absorbance was measured at 595 nm using a SPECTROstar BMG microplate reader [16].

### 2.5. Cell Culture and Cell Treatments

The human neuroblastoma SH-SY5Y cell line was cultured in DMEM (Lonza, Walkersville, USA) supplemented with 10% heat-inactivated FBS and 1% penicillin-streptomycin solution in a humidified atmosphere with 5% CO_2_ at 37 °C. The SH-SY5Y cells were pretreated with different concentrations of *Moringa oleifera* methanol extracts (5, 10 and 25 µg/mL) for 24 h before hydrogen peroxide exposure to induce oxidative stress (100 µM, 1 h).

### 2.6. Cell Viability

Cell viability was measured using the colorimetric MTT assay [17]. MTT solution (2 mg/mL) (Sigma-Aldrich) was added for 1 h and then formazan crystals were dissolved in DMSO. Absorbance was measured at 550 nm using a SPECTROstar BMG microplate reader. Cell viability was calculated as a percentage of no extract cells (100%). 

### 2.7. Intracellular Reactive Oxygen Species (ROS)

Intracellular ROS production was measured by dichlorofluorescein assay [18]. After cell treatments, SH-SY5Y cells were incubated in the fluorogenic dye 2,7-di-chloro-dihydrofluorescein diacetate (DCFH-DA) (0.01 M) (Sigma-Aldrich) for 30 min at 37 °C. ROS convert DCFH-DA into 2’-7’dichlorofluorescein (DCF). The intensity of the fluorescent signal was measured in a microplate fluorescence reader (FL×800, Bio-TekInstrumentation) at a λ excitation of 480 nm and λ emission of 580.

### 2.8. Thiobarbituric Acid Reactive Species (TBARS)

Lipid peroxidation content was determined by TBARS assay [19]. Total extracts (50 μL) were mixture with TBA-TCA-HCl (100 μL) and then boiled at 100 °C for 10 min. Absorbance was read at 535 nm using a SPECTROstar Omega microplate reader. 

### 2.9. Glutathione Assay

Total cell extracts were incubated with O-phthalaldehyde (OPT) (Sigma-Aldrich) and sodium phosphate buffer for 15 min. Fluorescence intensity was measured at an excitation wavelength of 528 nm and emission wavelength of 485 nm using a FLUOstar Omega microplate reader [20].

### 2.10. Antioxidant Enzymes Activity

For preparation of total cell extracts, pellets were resuspended in lysis buffer (TRIS 25 mM, NaCl 150 mM, EDTA 1 mM and Triton×100 (0.1%), pH 7.4) with antiproteases (phenylmethylsulfonyl fluoride, pepstatin and leupeptin). 

#### 2.10.1. Catalase Activity (CAT)

Total cell extracts were incubated with hydrogen peroxide (15 mM) and absorbance was measured at 240 nm for 1 min using a SPECTROstar Omega microplate reader [21]. 

#### 2.10.2. Superoxide Dismutase Activity (SOD)

Total cell extracts were incubated with pyrogallol (0.15 mM) dissolved in HCl (10 mM) and Tris−DTPA (pH 8.2). Absorbance was measured at 420 nm for 1 min using a SPECTROstar Omega microplate reader [22].

#### 2.10.3. Glutathione Peroxidase Activity (GPx)

Total cell extracts were incubated with EDTA, GSH, glutathione reductase, NADPH, sodium azide and phosphate buffer for 4 min. Then hydrogen peroxide was added and absorbance was measured at 340 nm for 3 min using a SPECTROstar Omega microplate reader [23].

### 2.11. Mitochondrial Membrane Potential

After treatments, cells were incubated with tetramethylrhodamine methyl ester (TMRM) (Gibco-Invitrogen, Grand Island, NY, USA) in Krebs medium and fluorescence was measured for 45 min at 37 °C with an emission wavelength and excitation wavelength of 549 nm and 573 nm, respectively, using a FLUOstar OPTIMA (BMG Labtech, Ortenberg, Germany) microplate reader. Then, oligomycin and trifluoromethoxy carbonylcyanide phenylhydrazone (FCCP) (Sigma-Aldrich) were added to induce mitochondrial depolarization. Fluorescence was measured for 15 min. The mitochondrial membrane potential value is the result of the difference between the maximum fluorescence and the baseline fluorescence [24]. 

### 2.12. Mitochondrial Calcium Levels

After treatments, cells were incubated with the cationic dye Rhod-2/AM (Gibco-Invitrogen) in Krebs normal calcium medium for 40 min. Then, cells were incubated in Krebs normal calcium medium for 30 min. Fluorescence was recorded for 4 min at 581 nm emission wavelength and 552 nm excitation wavelength using a FLUOstar OPTIMA (BMG Labtech, Ortenberg, Germany) microplate reader. Finally, the calcium ionophore A23187 (Sigma-Aldrich) was added, and fluorescence was measured for 15 min. Mitochondrial calcium levels were calculated as the ratio between the maximum fluorescence signal after the calcium ionophore A23187 addition and the basal fluorescence [25].

### 2.13. Cytosolic Calcium Levels

After treatments, cells were incubated with Indo-1/AM (Gibco-Invitrogen) in Krebs medium for 45 min at 37 °C. Then, cells were maintained in dye-free Krebs medium for 15 min at 37 °C. Fluorescence was measured for 4 min at 37 °C with an emission wavelength and excitation wavelength of 410 nm and 350 nm, respectively, using a FLUOstar OPTIMA microplate reader. Following this, ionomycin and MnCl_2_ were added, and fluorescence was measured under identical conditions for 8 min and 4 min, respectively. Cytosolic calcium levels were measured using the following formula: [Ca^2+^] i =Kd x [F−Fmin]/[Fmax−F], where Kd is the dissociation constant for Indo-1; F is the fluorescence signal for samples; Fmax is the maximum fluorescence signal after ionomycin addition and Fmin is calculated using this formula: Fmin = AF + 1/12 × (Fmax−AF), AF being the minimum fluorescence after adding MnCl_2_ [26].

### 2.14. Statistical Analysis

Data are presented as mean ± standard deviation. Statistical analysis was performed by SigmaPlot version 11.0 (Systat Software Inc., San Jose, CA, USA). Data were analyzed by one-way analysis of variance (ANOVA) with Tukey’s test. Statistical significance was set at *p* < 0.05. 

## 3. Results and Discussion

Although *Moringa oleifera* has demonstrated numerous therapeutic activities, studies on its neuroprotective activity are still limited. The aim of the present work is to investigate the potential neuroprotective role of methanol extracts of *Moringa oleifera* leaf powder on antioxidant/ROS imbalance and mitochondrial regulation in a hydrogen peroxide-induced oxidative stress model in human neuroblastoma cells.

### 3.1. Nutritional Value 

The nutritional value of the leaves of *Moringa oleifera* is shown in Table 1. The content of proteins is over 25%; this value is higher than that of other vegetables with high-protein content (potato, pumpkin) and is similar to other high-protein foods (i.e., milk, eggs and meat) [27]. Moreover, *Moringa oleifera* has a low–medium content in fat (over 10%) and carbohydrates (28.5%). Furthermore, moringa leaf powder has 12% of dietary fiber, 4% of ashes and 0.52% of moisture. Comparing the results of the nutritional composition of leaf powder of *Moringa oleifera* of our study with other previously published ones, there are certain differences in carbohydrates (25.5% versus 44.4%), fat (10% versus 7.1%) and ash content (4% *versu*s 10.9%) [28]. It is especially worth highlighting the total carotenoid content (1.10 mg β-carotene), vitamin B1 (326 µg/100 g) and vitamin C (15.2 mg/100 g). Moringa leaves have more vitamin A than carrots and more vitamin C than oranges [29].

### 3.2. In-Vitro Antioxidant Assays

The results of the in-vitro antioxidants assays and phenolic content are shown in Table 2. The yield of moringa leaf powder was similar for both methanol extraction and acetone extraction (6.38% and 6.66%, respectively). The in-vitro antioxidant activity of methanol and acetone extracts was measured using DPPH assay, ORAC method and FRAP assay. These methods differ in the type of reaction: hydrogen atom transfer (HAT)-based assays and electron transfer (ET)-based assays. HAT assays measure the ability of antioxidants to scavenge free radicals by H-atom donation [i.e., ORAC assay]. ET assays measure the ability of antioxidants to reduce an oxidant, which changes color when reduced [i.e., FRAP and DPPH]. The results of the present work showed that methanol extract has significant antioxidant activity than acetone extract. Hence, DPPH values were 44.89 µg/mL and 305.44 µg/mL, ORAC value 8360 µmol TE/100 g sample and 866.1 µmol TE/100 g sample and FRAP value 59.32 µmol Fe^2+^ eq/g sample and 27.91 µmol Fe^2+^ eq/g sample for methanol and acetone extracts, respectively.

For the additional characterization of moringa extracts, total phenolic content was evaluated using Folin–Ciocalteu method. The amount of total phenolic content was high in methanol extract (2.17 mg gallic acid/g extract) than in acetone extract (0.88 mg gallic acid/g extract). The variations in the phenolic content are attributed to the time of harvesting of the leaves, the growing conditions, and the solvent of the extraction. Hence, the more mature the moringa leaves, the higher the phenolic content [30]. There are different phenolic compounds identified in *Moringa oleifera* leaves such as rutin, quercetin, isoquercetin, astragalin, kaempferol, apigenin, luteolin, genistein, myricetin, and vicenin-2, among others [8]. Epidemiological studies have shown a relationship between polyphenol-rich foods and a lower risk of suffering neurodegenerative diseases [31].

### 3.3. Effect on Cell Viability and Cell Morphology 

Since the methanol extract of moringa leaf powder had greater antioxidant activity, we selected this extract to evaluate its potential neuroprotective activity in a cellular model of oxidative stress induced by hydrogen peroxide. Oxidative stress is one major pathway contributing to neurodegeneration. The brain has some characteristics that make it especially susceptible to oxidative stress, including high rate of oxygen consumption, low catalase content, neurotransmitters (dopamine) that can auto-oxidize and high content in redox active transition metals [32]. Medicinal plants with antioxidants properties can slow or stop the neurodegenerative processes through scavenging free radicals, metal chelation or upregulating endogenous antioxidant system. Polyphenolic compounds can pass across blood-brain barrier [33]. Earlier studies demonstrated that *Moringa oleifera* could have a neuroprotective effect against oxidative stress. Hence, the methanol leaf extract improved the homocysteine and AF64A induced oxidative stress, cognitive impairments and Aβ pathology in rats, being a promising candidate for Alzheimer’s disease [34,35]. Moreover, the methanol extract of moringa leaves (250 mg/kg, 300 mg/kg) attenuated lead and aluminum-induced cerebral damage in rats by reducing oxidative stress, inflammation and apoptosis and by improving neurohistopathology [36,37]. Compounds identified as potential neuroprotective agents for moringa include isothiocyanate isolated from seeds [38].

**Figure 1:**
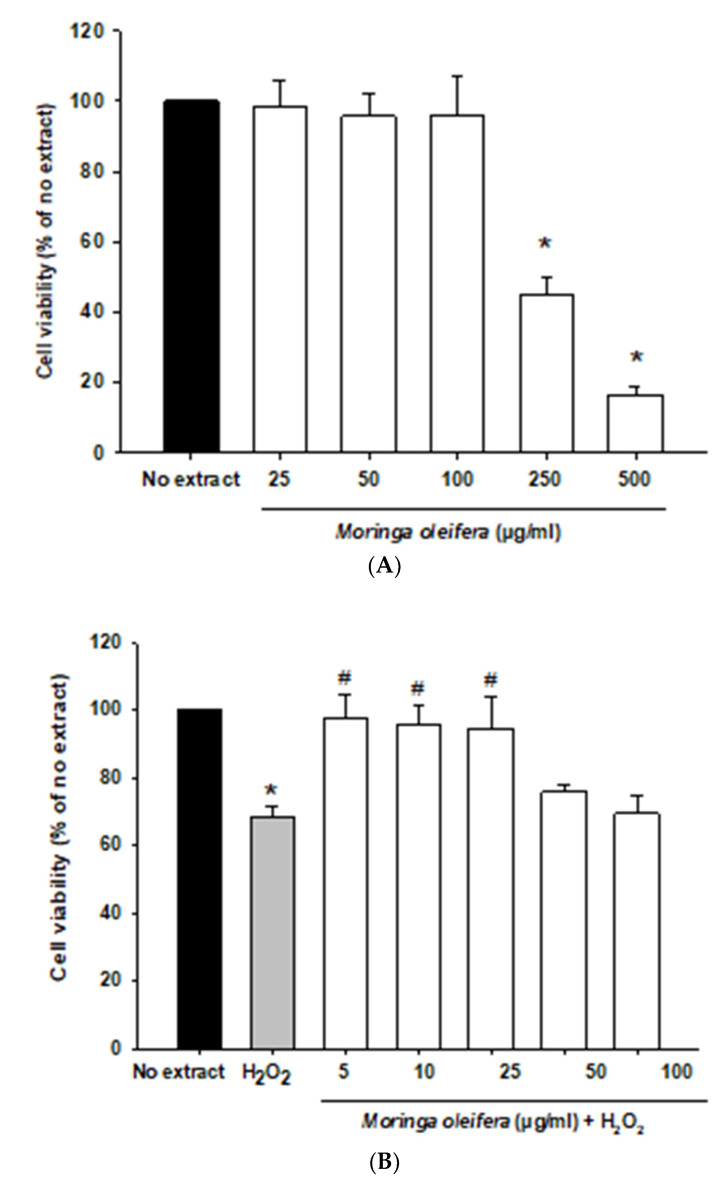

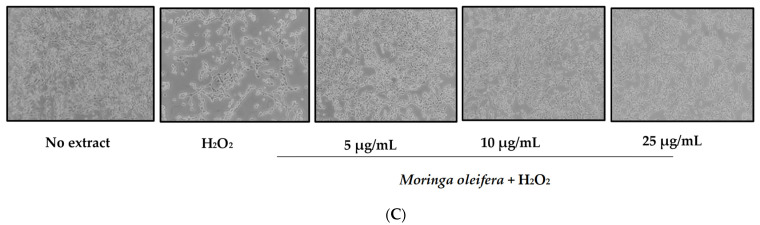
Effect of methanol extracts of *Moringa oleifera* leaf powder on cell viability and cell morphology. (**A**) Effect of moringa on SH-SY5Y cell viability. Cells were treated with moringa from 5 to 500 µg/ml for 24 h. (**B**) Protective effect of moringa in a hydrogen peroxide-induced oxidative stress model. Cells were pretreated with moringa from 5 to 100 µg/ml for 24 h before 100 µM H_2_O_2_ for 1 h. (**C**) Effect of moringa on SH-SY5Y cell morphology. * *p* < 0.05 versus no extract, ^#^ *p* < 0.05 versus H_2_O_2._

In the current study, we initially investigated the effect of different concentration of *Moringa oleifera* methanol leaves extracts (from 5 to 500 µg/mL) on the human neuroblastoma SH-SY5Y cells. This cell line is widely used as a dopaminergic neuronal model for common neurodegenerative diseases such as Alzheimer’s disease, Parkinson’s disease, and Amyotrophic lateral sclerosis. SH-SY5Y cells express dopamine transporter and the enzymes tyrosine hydroxylase and dopamine-beta-hydroxylase [39]. As shown in Figure 1A, all assayed concentrations were non-toxic except for 250 and 500 µg/mL which significantly decreased cell viability. Next, an in-vitro oxidative stress model was established with hydrogen peroxide (100 µM, 1 h). Hydrogen peroxide passes across membranes using aquaporin-3 [40]. Hydrogen peroxide significantly reduced cell viability by around 32% compared to no extract cells. However, pretreatments with 5, 10 and 25 µg/mL significantly increased the viability of cells by over 25–30% compared to hydrogen peroxide-treated cells, as shown in Figure 1B. Moreover, hydrogen peroxide modified morphology towards round cells. *Moringa oleifera* extracts prevented these alterations in neuroblastoma cells morphology (Figure 1C). Previous studies demonstrated that the aqueous extract of moringa protected cell viability and ameliorated cytotoxicity against cadmium in colon and kidney cells [41].

### 3.4. Effect on Intracellular ROS Production

**Figure 2:**
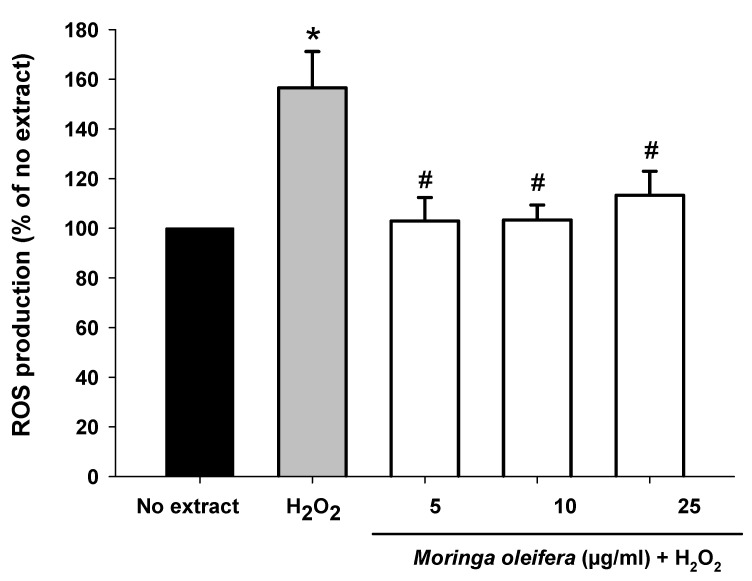
Effect of methanol extracts of *Moringa oleifera* leaf powder on intracellular ROS overproduction. Cells were pretreated with moringa (5, 10 and 25 µg/ml) for 24 h before 100 µM H_2_O_2_ for 1 h. * *p* < 0.05 versus no extract, ^#^ *p* < 0.05 versus H_2_O_2._

The most cytoprotective and non-toxic concentrations (5, 10 and 25 µg/mL) of *Moringa oleifera* methanol extracts were selected for subsequent experiments. Hydrogen peroxide can be converted into hydroxyl radical (HO•) via Fenton reaction [42]. The intracellular production of ROS was measured using the DCFHA-DA assay. As shown in Figure 2, hydrogen peroxide significantly increased by 56% compared to no extract cells. However, pretreatments with the three assayed concentrations of *Moringa oleifera* significantly reduced hydrogen peroxide-induced ROS overproduction. Similar results were observed for moringa leaf flavonoids in bovine mammary epithelial cells against hydrogen peroxide and the compound 1-O-(4-hydroxymethylphenyl)-α-L-rhamnopyranoside from *Moringa oleifera* seeds against carbon against CCl_4_ [43,44]. 

### 3.5. Effect on Oxidative Stress Markers

**Figure 3:**
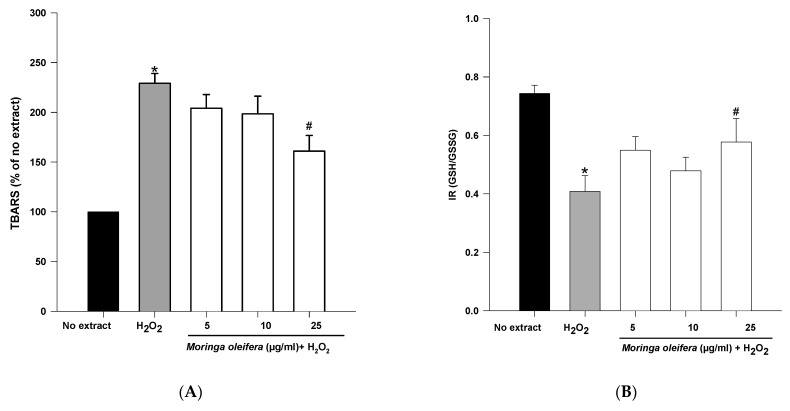
Effect of methanol extracts of *Moringa oleifera* leaf powder on (**A**) lipid peroxidation and (**B**) Index Redox (GSH/GSSG). Cells were pretreated with moringa (5, 10 and 25 µg/ml) for 24 h before 100 µM H_2_O_2_ for 1 h. * *p* < 0.05 versus no extract, ^#^ *p* < 0.05 versus H_2_O_2._

An overproduction of ROS to the detriment of endogenous antioxidants leads to oxidative damage to biomolecules. Lipid peroxidation and GSH content are clinically significant biomarkers of oxidative stress in humans [45]. The double bonds of polyunsaturated fatty acids are highly vulnerable to ROS attack and consequently to lipid peroxidation. Malondialdehyde (MDA) is one the most investigated end product of lipid oxidation [42]. As shown in Figure 3A, hydrogen peroxide significantly increased the peroxidation of lipids by 229%. However, pretreatments with methanol extract of moringa leaf powder at 25 µg/mL significantly reduced TBARS levels (160% compared to 229% of hydrogen peroxide). Therefore, moringa leaf extract has a potent preventive effect against the peroxidation of lipids. As shown above, the methanol extract of moringa has scavenging capacity, so the effect on lipid peroxidation can be attributed to a direct action on free radicals process [33].

Reduced glutathione is the major antioxidant compound in mammalian cells. The index redox (GSH/GSSG) was significantly decreased in hydrogen peroxide (IR = 0.41) compared to no extract cells (IR = 0.74). On the other hand, methanol extract of moringa at 25 µg/mL significantly increased IR to 0.578 compared to hydrogen peroxide-treated cells. 

### 3.6. Effect on Antioxidant Enzymes Activities

**Figure 4:**
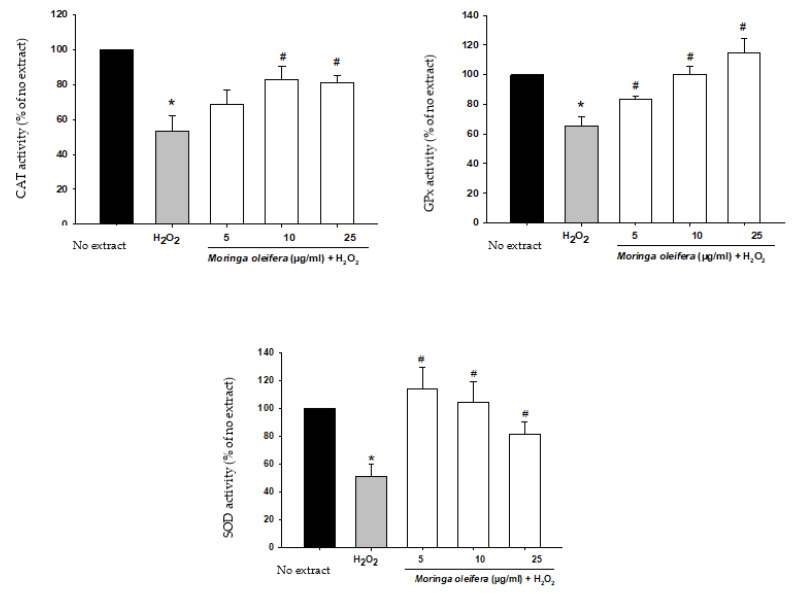
Effect of methanol extracts of *Moringa oleifera* leaf powder on antioxidant enzyme activity. Cells were pretreated with moringa (5, 10 and 25 µg/ml) for 24 h before 100 µM H_2_O_2_ for 1 h. * *p* < 0.05 versus extract, ^#^ *p* < 0.05 versus H_2_O_2._

The antioxidant enzymatic defense system plays a key role against free radicals. The enzymes catalase (CAT), superoxide dismutase (SOD) and glutathione peroxidase (GPx) constitute the first defense system against oxidative stress. Superoxide dismutase (SOD) converts the superoxide anion radical into hydrogen peroxide. Catalase (CAT) and glutathione peroxidase (GPx) catalyze the decomposition of hydrogen peroxide to water (GPx) and to water and oxygen (CAT) [46]. In Figure 4, our study demonstrated that there is a significant decrease in the activity of these antioxidant enzymes by 53% for CAT, 51% for SOD and 65% for GPx compared to no extract cells (100%). However, pretreatments with moringa extracts reversed this impairment in the defensive system. For catalase, there was a significant increase at 10 and 25 µg/mL (82.9% and 81.2%). For SOD and GPx, the three assayed concentrations markedly improved the activity of these enzymes. The ability of moringa to increase antioxidant enzymatic activity suggests that their polyphenols have a direct antioxidant effect [33].

### 3.7. Effect on Mitochondrial Regulation

**Figure 5:**
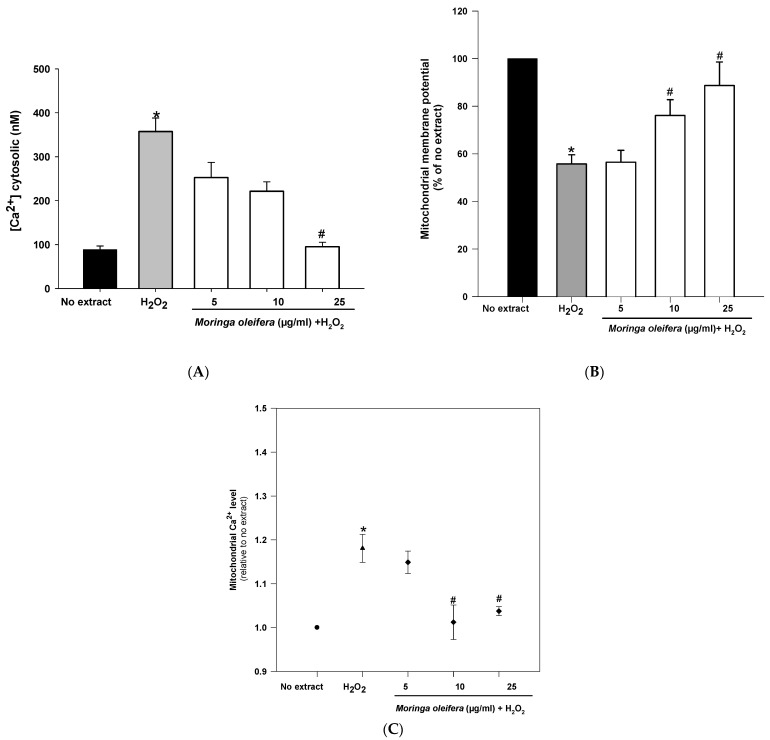
Effect of methanol extracts of *Moringa oleifera* leaf powder on (**A**) mitochondrial membrane potential (**B**) levels of cytosolic calcium and (**C**) levels of mitochondrial calcium. Cells were pretreated with moringa (5, 10 and 25 µg/mL) for 24 h before 100 µM H_2_O_2_ for 1 h. * *p* < 0.05 versus no extract, ^#^ *p* < 0.05 versus H_2_O_2._

Mitochondria are double membrane-bound cell organelles presented in almost all cell eukaryotes. Mitochondria are involved in energy production [47]. The increased ROS generation can be associated with mitochondrial dysfunction. Mitochondria is a key target for the prevention and treatment of those oxidative stress-related neurodegenerative diseases [48]. As shown in Figure 5A, hydrogen peroxide reduced significantly mitochondrial membrane potential (MMP) by 55% compared to no extract cells (100%). However, pretreatments with 10 µg/mL and 25 µg/mL significantly increased MMP to 76% and 88%, respectively. Regarding calcium cytosolic, there was a significant increase (357 nM) after hydrogen peroxide-treated cells compared to no extract cells (88 nM). However, the pretreatment with 25 µg/mL almost reduced cytosolic calcium levels to no extract levels (95 nM) (Figure 5B). Furthermore, hydrogen peroxide significantly increased mitochondrial calcium level (1.18 relative to no extract). This increase in mitochondrial calcium level was markedly reduced after pretreatments with 10 µg/mL and 25 µg/mL of moringa extracts (1.01 and 1.03 relative to no extract, respectively) (Figure 5C). Hydrogen peroxide interacts with mitochondrial structure and function, leading to an impaired calcium level and a mitochondrial potential deficiency [49]. Altered calcium levels can affect mitochondrial function and integrity (i.e., depletion of mitochondrial DNA, release of cytochrome C, ultrastructural lesions, morphological changes) as well as to promote the production of more ROS (i.e., peroxidation of cardiolipin), contributing to the pathogenesis of many common neurodegenerative diseases [50]. The methanol extract of moringa leaves exerts a mitochondrial protective role in neurons, showing for the first time that the mitochondria is the target of action for this medicinal plant. 

The major polyphenols identified in methanolic extract of leaves of *Moringa oleifera* include quercetin, kaempferol, apigenin and gallic acid [8]. Previous studies have demonstrated that, after intestinal absorption, polyphenols can reach bloodstream concentration to exert their action [51]. Particularly, these polyphenols presented in moringa have been detected and quantified in blood (quercetin at a concentration of 0.0723 ± 0.0810 µM; kaempferol at a concentration of 0.0579 ± 0.0609 µM; apigenin at a concentration of 0.0106 ± 0.0123 µM and gallic acid at a concentration of 0.007 ± 0.003 µM) [52,53,54]. Additionally, Nair et al. (2020) have recently demonstrated that compounds presented in moringa extracts are able to pass across Blood-brain-barrier (BBB) and to reach its pharmacological target [55]. Regarding the safe toxicological profile of moringa, doses of up to 2000 mg/kg of methanol and aqueous leaf extract were safe in animal studies [56,57]. 

Conversely, gallic acid is one of the most abundant phenolic compounds identified in the leaf extract [58]. Previous studies demonstrated that pretreatments with gallic acid protected SH-SY5Y cells against 6-Hydroxydopamine (6-OHDA) induced damage by attenuating mitochondrial dysfunction, reducing intracellular ROS level, and inhibiting apoptotic cell death [59]. Moreover, pretreatments with gallic acid also exerted a potent protection in a hydrogen peroxide-induced oxidative stress model in SH-SY5Y cells by decreasing intracellular ROS production, increasing REDOX activity, and inhibiting caspase-3 activation [60]. Apart from its neuroprotective effect in oxidative stress models in SH-SY5Y cells, gallic acid and its derivatives have shown to inhibit the aggregation of several amyloid proteins associated with the pathological process of many neurodegenerative diseases. Hence, the oral administration of gallic acid improved memory in an APP/PS1 transgenic mouse model by reducing Aβ1-42 aggregation [61]. Moreover, gallic acid also inhibited the insulin amyloid fibril formation in an in-vitro model [62]. Studies on the relationship between structure and inhibitors of amyloid formation have revealed that gallic acid owes its activity to both aromatic and hydroxyl groups and the presence of galloyl moiety [62,63]. In addition to gallic acid, epigallocatechin-3-gallate has shown to reduce Abeta generation in murine neuron-like cells (N2a) transfected with the human “Swedish” mutant amyloid precursor protein (APP) and in primary neurons derived from Swedish mutant APP-overexpressing mice (Tg APPsw line 2576) as well as to reduce the intensity of pancreatic amyloid fibrils in human islet amyloid polypeptide (hIAPP) in trasgenic mice [64,65]. Epigallocatechin-3-gallate exerts its action by binding transthyretin (TTR) and maintaining it in a non-aggregated soluble form [65,66,67,68]. Therefore, in our study, the neuroprotective effect of the methanol extract of moringa may be partly due to gallic acid and its derivatives. 

## 4. Conclusions

In conclusion, the current work provides preliminary evidence of the antioxidant-mediated neuroprotective effects of the methanol extract of *Moringa oleifera* leaf powder. Moringa has shown to exert a protective effect against hydrogen peroxide in neuroblastoma cells by reducing ROS overproduction and lipid peroxidation levels, by increasing GSH content and antioxidant enzymes activity and by avoiding mitochondrial dysfunction. The most active concentration was 25 µg/mL. This work supports and deepens the therapeutic properties of a nutritionally rich plant. Particularly, this research delves into the molecular mechanisms by which *Moringa oleifera* methanol extract exerts its neuroprotective effect. These promising results create the need for in-vivo studies and even clinical trials to validate the physiological relevance of our in-vitro data. 

## Figures and Tables

**Table 1:** Nutritional value of *Moringa oleifera* leaf powder.

	*Moringa oleifera* Leaf Powder
Ashes (%)	4.45 ± 0.33
Carbohydrates (%)	28.50 ± 0.45
Dietary fiber (%)	11.83 ± 1.19
Energy (kcal/100 g)	324.4 ± 2.89
Fat (%)	10.42 ± 0.63
Moisture (%)	0.52 ± 0.05
Proteins (%)	25.02 ± 0.37
Total carotenoids (mg β-carotene)	1.108 ± 0.12
Vitamin B1 (µg/100 g)	326.4 ± 1.28
Vitamin C (mg/ 100 g)	15.2 ± 0.78

Data are expressed as mean ± standard deviation (n = 3).

**Table 2:** In-vitro assays for antioxidant activity evaluation and phenolic content of methanol and acetone extracts of *Moringa oleifera* leaf powder.

Extracts of *Moringa oleifera* Leaf Powder	Yield of Extract (% *w*/*w*)	DPPH EC_50_ (µg/mL)	ORAC (µmol TE/100 g Sample)	FRAP (µmol Fe^2+^ eq/g Sample)	Total Phenol Content (mg gallic acid/g extract)
Methanol extract	6.38% ± 0.47	44.89 ± 1.01 *	8360 ± 0.05 *	59.32 ± 0.29 *	2.17 ± 0.03 *
Acetone extract	6.66% ± 0.09	305.44 ± 8.21	866.1 ± 0.01	27.91 ± 1.90	0.88 ± 0.005

Data are expressed as mean ± standard deviation (n = 3). * *p* < 0.05 versus acetone extract.

## Data Availability

Not applicable.
